# Real-time robustness evaluation of regression based myoelectric control against arm position change and donning/doffing

**DOI:** 10.1371/journal.pone.0186318

**Published:** 2017-11-02

**Authors:** Han-Jeong Hwang, Janne Mathias Hahne, Klaus-Robert Müller

**Affiliations:** 1 Department of Medical IT Convergence Engineering, Kumoh National Institute of Technology, Gyeongbuk-do, Gumi, Republic of Korea; 2 Neurorehabilitaiton Systems Research Group, Department of Trauma Surgery, Orthopedic Surgery and Hand Surgery, Universiy Medical Center Goettingen, Goettingen, Germany; 3 Machine Learning Group, Berlin Institute of Technology (TU Berlin), Berlin, Germany; 4 Department of Brain and Cognitive Engineering, Korea University, Seoul, Republic of Korea; 5 Max Planck Institute for Informatics, Stuhlsatzenhausweg, Saarbrücken, Germany; University of Illinois at Urbana-Champaign, UNITED STATES

## Abstract

There are some practical factors, such as arm position change and donning/doffing, which prevent robust myoelectric control. The objective of this study is to precisely characterize the impacts of the two representative factors on myoelectric controllability in practical control situations, thereby providing useful references that can be potentially used to find better solutions for clinically reliable myoelectric control. To this end, a real-time target acquisition task was performed by fourteen subjects including one individual with congenital upper-limb deficiency, where the impacts of arm position change, donning/doffing and a combination of both factors on control performance was systematically evaluated. The changes in online performance were examined with seven different performance metrics to comprehensively evaluate various aspects of myoelectric controllability. As a result, arm position change significantly affects offline prediction accuracy, but not online control performance due to real-time feedback, thereby showing no significant correlation between offline and online performance. Donning/doffing was still problematic in online control conditions. It was further observed that no benefit was attained when using a control model trained with multiple position data in terms of arm position change, and the degree of electrode shift caused by donning/doffing was not severely associated with the degree of performance loss under practical conditions (around 1 cm electrode shift). Since this study is the first to concurrently investigate the impacts of arm position change and donning/doffing in practical myoelectric control situations, all findings of this study provide new insights into robust myoelectric control with respect to arm position change and donning/doffing.

## 1. Introduction

There are a large number of upper-limb amputees who are restricted in conducting daily life activities, such as dressing, eating, and body care. To restore their hand functions at least partly, many efforts have been devoted to the development of both active prostheses and their control algorithms using electromyographic (EMG) signals. However, although the current state-of-the-art prosthetic hand devices allow even for individual finger movements (e.g., Touch Bionics’s i-LIMB), so far no myoelectric control algorithm is available, which fully follows the advance in mechatronics [1].

Pattern recognition techniques have been most actively studied to develop control algorithms for electrically powered hand prostheses [2–4], and demonstrated the excellent performance of discriminating different hand/wrist motions (i.e., > 95% for 10 classes) [5–10]. However, most pattern recognition approaches have an inherent limitation that only one prosthetic function can be controlled at a time due to their sequential and ON/OFF control manners. Such control strategies make it impossible to perform natural hand movements consisting of continuous and simultaneous activation of multiple DoFs. To provide a proportional control in pattern-recognition-based myoelectric prostheses, the classical approach can be extended by an estimation of the contraction level, which is combined with the classifier output [11, 12]. A recent study demonstrated excellent control performance with such an approach [13]. Also, some studies have introduced novel pattern recognition schemes which classify combined motions for simultaneous prosthesis control [14–18], and one of them showed the possibility of an independent proportional control of a selected motion [15]. One drawback of the new approach is that the total number of classes is dramatically increased because all possible class-combinations are regarded as new classes. This increases not only the (computational) complexity of the classification algorithm, but may also become a practical problem when training data for each class combination has to be recorded.

Recently, regression-based approaches have attracted researchers’ attention as a possible alternative to the above classification schemes [2, 3]. This is because regression algorithms provide independent simultaneous and proportional control information for myoelectric prostheses with multiple DoFs, which enables more natural and intuitive prosthesis control. In the last years, offline studies have introduced various linear and non-linear regression methods [1, 19–24] and shown the possibility of applying regression techniques in controlling upper-limb prostheses simultaneously and proportionally. Most recent studies have further proven the applicability of regression approaches in online control scenarios that mimic real situations of myoelectric prosthesis control [25–33]. However, their clinical reliability and robustness have not fully been investigated so far.

Despite the great advances in the development of myoelectric control algorithms based on pattern recognition and regression techniques, most commercially available prosthetic hands still use the direct control method introduced more than a half century ago [3]. The conventional control approach can actuate only a single DoF of a prosthesis at a time by comparing EMG amplitudes measured from two different muscle locations (e.g., extensor and flexor), and uses heuristics such as a co-contraction strategy to switch between available DoFs. The inconsistency between academic achievements and industrial products (see [2]) can be explained by the fact that research results are generally obtained without considering robustness and nonstationarity issues [34, 35] that significantly affect the myoelectric control performance in practical conditions, thereby making it hard to directly transfer advanced academic achievements to the industry. It was not until recently that myoelectric control based on pattern recognition became commercially available for the first time [36], and a clinical trial was performed with a bilateral upper limb amputee [37]. More recent results showed positive outcomes of pattern-recognition-based myoelectric control in clinical studies [38, 39].

Arm position change and prosthesis donning/doffing are important factors associated with robust myoelectric control because they most frequently occur during use of a prosthetic hand and change important environmental conditions in myoelectric control. In particular, arm position change mainly causes muscle displacement due to altered joint angles, gravity muscle contractions, and compression at electrode sites in a prosthetic socket. Donning/doffing potentially results in electrode shifts and altered electrode impedances. Previous studies examined the impacts of electrode shift [20, 40–42] and arm position change [21, 43–47] on myoelectric control performance, and showed that changing the control conditions negatively affects the performance of both pattern recognition [40–46] and regression [20, 21] algorithms. Some of them suggested potential solutions to alleviate the adverse effect of the condition changes in myoelectric control, such as incorporating the data collected from shifted electrode positions into training data [42] and using accelerometers to detect arm position changes [43, 44, 47].

On the other hand, a recent study investigated the relationship between offline and online (real-time) myoelectric control performance with three regression algorithms [48]. The main finding of this study was, surprisingly, that there is little correlation between offline and online performance indices. All subjects participated generally achieved good online performance even though some of them did not show reliable offline performance (< 40%), underlining that precise myoelectric control in practical closed-loop conditions can be achieved by the interaction between the user and a myoelectric controller through proper feedback regardless of offline performance. The results also indicate that an offline performance index is imperfect to accurately predict real myoelectric controllability attained in practical situations. In this respect, the impacts of the representative condition changes (arm position change and donning/doffing) in myoelectric control should be also examined in online environments to obtain a realistic characterization of myoelectric control and to find promising solutions to relieve their adverse impact. However, most of relevant results have been obtained from offline experiments [20, 21, 42–46]. Only a few studies using pattern-recognition schemes reported the impacts of arm position change [49] and channel shift [40, 41] on online myoelectric controllability, and there are little relevant results for regression-based myoelectric control algorithms that are promising alternatives to pattern-recognition techniques.

In the present study, we investigated how the two important factors, arm position change and donning/doffing, affect myoelectric controllability based on a regression approach in online control conditions that reflect practical myoelectric control situations. To this end, the impacts of arm position change and donning/doffing were independently and concurrently tested on three different arm positions for which we designed a two-dimensional target acquisition task that adopts a linear regression method allowing simultaneous and proportional myoelectric control. Seven performance metrics were employed to evaluate the changes in real-time myoelectric controllability in terms of arm position change and donning/doffing, and statistical tests were appropriately conduced for each performance measure. The rest of the paper is organized as follows. The detailed experimental procedures and analysis methods are described in section 2, followed by the experimental results in section 3. The obtained results are discussed and summarized in section 4.

## 2. Methods

### 2.1. Subject

Fifteen normally limbed subjects (11 males and 4 females aged 25–53 years; all right handed) were recruited by personally contacting graduate students in the Machine Learning Laboratory of Technical University of Berlin, and via a local community website in Berlin, Germany between April and May of 2014. One person with congenitally deficient upper limb (male, 41 years) whose right forearm terminates at wrist level was recruited via personal contact in Göttingen, Germany. All recruited subjects participated in this study and no subjects were dropped out for data analysis. Six normally limbed subjects took part in preliminary experiments, four of which also took part in main experiments. Fourteen subjects including the congenital amputee subject took part in main experiments. None of them had a previous history of neuromuscular disorders that might influence experimental results. The fully detailed summary of our research goal and experimental procedures were explained to each subject, and they signed written consents before the experiment. Adequate reimbursement was given to them for their participation after the experiment. This study was approved by the ethics committee of Charité-University of Berlin Medicine (approval number EA4/085/11), and all experiments were conducted in accordance with the declaration of Helsinki.

### 2.2. EMG data acquisition

**Fig 1:**
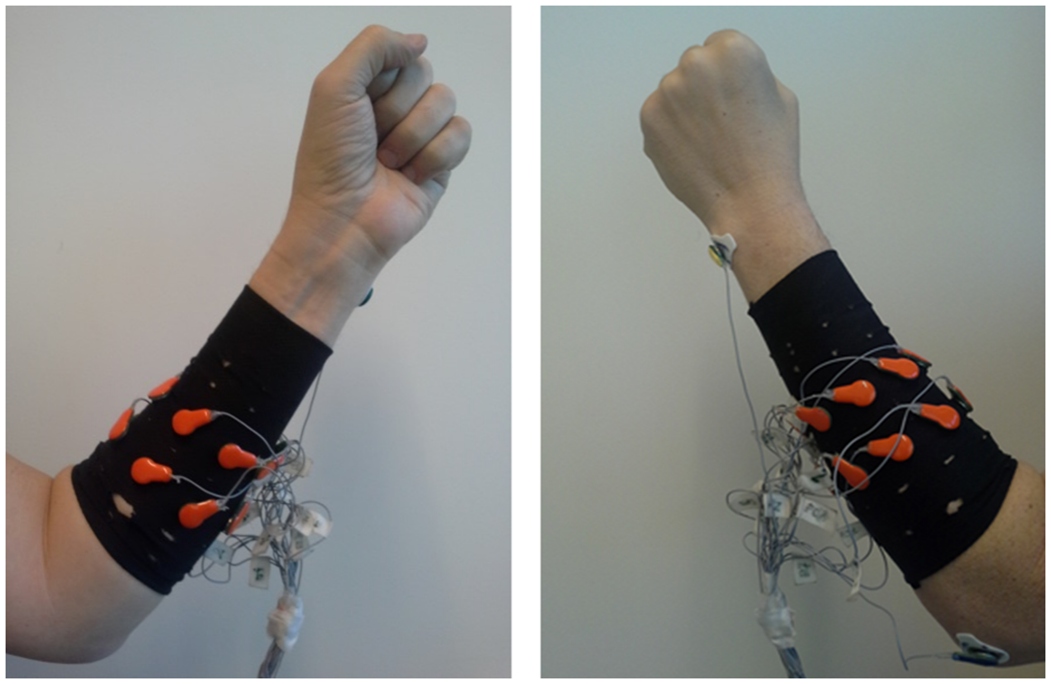
Forearm of a normally limbed subject wearing the textile hose including 16 electrodes.

EMG signals were recorded using a 16 channel biosignal amplifier (g.USBamp, g.tec Inc., Graz, Austria) with a dry active electrode system (g.SAHARA). Since the g.SAHARA dry electrode system was originally designed for recording brain signals, it was not adequate to measure EMG signals on the forearm. Thus, pin-type g.SAHARA electrodes were replaced with custom made flat-type dry electrodes (12 mm diameter) [31]. For fast and easy experiment setup, the new electrodes were integrated into a custom-made stretchable textile hose, where they were equidistantly mounted along two circles with an inter-electrode distance of 35 mm. In the experiment, the hose was placed on the left forearm of the able-bodied subjects and the affected right forearm of the subject with congenital limb deficiency. The position of the electrodes integrated in the hose was fitted around the thickest part of the forearm, which was approximately at 1/3 of the distance from the elbow to the wrist. The reference and ground electrodes were placed around the olecranon process and the styloid process of the ulnar, respectively. Fig 1 shows the configuration of the 16 electrodes integrated into the textile hose. The surface EMG signals were acquired at a sampling rate of 1200 Hz using a 24-bit A/D converter. All EMG data used in this study are available from the following website: http://doc.ml.tu-berlin.de/AMYO.

### 2.3. Real-time myoelectric system and its control algorithm

**Fig 2:**
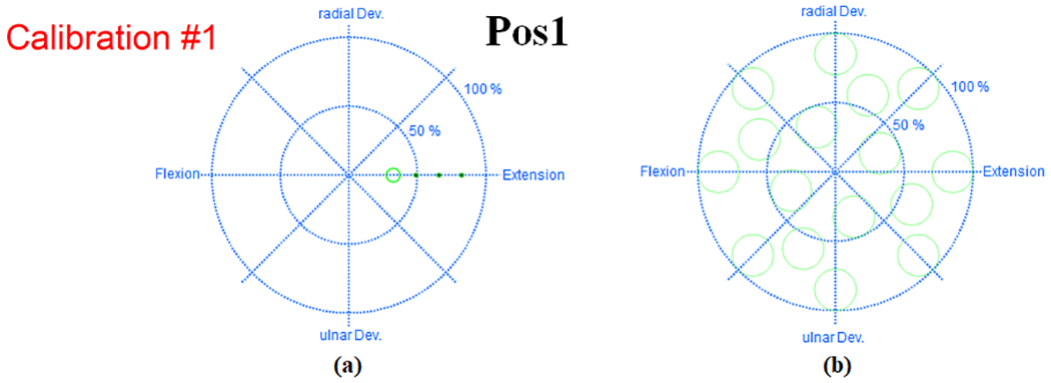
Graphical user interface (GUI) of the implemented online myoelectric control system. (a) Screen shot of the calibration run. At the beginning of the calibration run, three small circles sequentially start moving to a predefined direction corresponding to one of the two DoF wrist movements (flexion-extension and radial-ulnar deviation), during which the subject is asked to wait for an upcoming a larger circle. The three small circles are introduced to give the subject a preparation time, and the larger circle is the actual visual target the subject should follow. When the larger circle appears and starts following the three small circles, the subject is prompted to make wrist contractions based on the position of the larger circle moving from the center (rest position) to the end point of the outer circle (full contraction). The time used for each direction is 8 s, which consists of 3 s movement from the center to the end position, 2 s dwell time at the end position, and 3 s returning movement to the center. The target circle (larger one) is sequentially presented to extension, radial deviation, flexion and ulnar deviation direction. (b) Location of all 16 targets used in the online evaluation run. At the beginning of the online test run, a pink cursor first appears in the center for 3 s, and then a target is randomly presented with a pure-tone beeping sound, which is the signal to start performing the task. To acquire a target, the cursor should be entered into the circle within a time limit and stayed for 1 s. The time limit is set to 5 s for the preliminary experiment and 10 s for the main experiment, considering task difficulty after arm position change and donning/doffing. After the subject hits or misses a target, the cursor is automatically returned to the center and a new target is given to the subject with the beeping sound. There is a 1-s pause between targets for the subject to prepare the next acquisition task. This procedure is iterated for all 16 targets. The targets are presented with differently randomized orders for each evaluation run and each subject.

To investigate the impacts of arm position change and donning/doffing in online control scenarios, a real-time myoelectric control system implemented in MATLAB^™^ was used in which a cursor on a computer monitor is controlled based on EMG signals in two-dimensional Cartesian space (see Fig 2 to see the system interface in advance). To control a cursor, two wrist DoFs were used in this study, which are wrist flexion/extension and radial/ulnar deviation. The wrist flexion-extension was mapped to the horizontal movement of the cursor, while the radial-ulnar deviation was mapped to the vertical movement of the cursor. The speed of the cursor was simultaneously and proportionally controlled in 2D space by the combinations of the two wrist DoF movements (velocity control). When no contraction was made (rest position), the cursor stayed at the current position. All data processing was performed using a window length of 200 ms and an increment of 40 ms, meaning that the cursor was updated every 40 ms (25 Hz update rate). This window length is within acceptable controller delays for prosthesis control [50].

#### 2.3.1. Preprocessing

A common average reference (CAR) spatial filter was applied to real-time EMG data to remove common noise components across the recording electrodes. To this end, the mean EMG values of all channels was calculated and then subtracted from those of each channel for every time point. The power-line interference and its harmonic components were also eliminated by using 50 Hz comb filters, and then the EMG data were bandpass filtered using a 4th order Butterworth filter between 30 Hz and 300 Hz to remove movement artifacts and high frequency noise.

#### 2.3.2. Feature extraction

Root-mean-square (RMS) values were calculated from the preprocessed EMG data of each channel, yielding a 16-dimensional feature-vector **x**(*t*) used for estimating continuous wrist movements in 2D space. As mentioned above, because the data processing was performed every 40 ms, a new 16-dimensional feature-vector **x**(*t*) was created every 40 ms. RMS features were successfully employed for simultaneous and proportional myoelectric control of multiple DoFs [25, 51, 52].

#### 2.3.3. Regression

A linear regression method was applied to each analysis window for decoding continuous wrist movements of the two DoFs **ŷ**(*t*) from the feature-vector **x**(*t*):

$$\hat{y}(t)\;=\;W^{T}x(t)+w_{0},$$ (1)

where **W** is a matrix of regression coefficients and the bias **w**_**0**_ is included in **W** by extending **x**(*t*) with the constant 1. The regression output **ŷ**(*t*) is a vector in two-dimensional space and represents the wrist angles estimated from the EMG features by linear regression. It will be used as input for real-time cursor control as described in section 2.3.4. To estimate the regression model **W**, a series of **x**(*t*) and **y**(*t*) were acquired in the calibration session described in the section 2.4.1, which are represented as **X** ∈ ℝ^***C***×***T***^ and **Y** ∈ ℝ^***D***×***T***^. Since the RMS features were extracted from 16 channels and two wrist DoFs movements were employed, *C* and *D* are sixteen and two, respectively. Also, *T* represents the number of samples in the training set, and thus the size of **X** and **Y** can be flexible depending on the experimental time. The simplest and most common approach for estimating the regression matrix is ordinary least squares (OLS) that minimizes the sum of squared residuals by which the solution can be obtained in a closed form:

$${W\;=\;(XX}^{T}{)}^{-1}XY^{T}$$ (2)

Once the regression estimator **W** is obtained, myoelectric cursor control information **ŷ**(*t*) is continuously estimated by simple multiplication of **W** and the feature vector **x**(*t*) acquired in real-time, as denoted in (Eq 1).

#### 2.3.4. Post-processing

The instantaneous regression output **ŷ** requires post-processing for smooth and reliable myoelectric control because it inherently contains undesired high-frequency components that appear as unstable cursor movement (e.g., jumping up and down), despite the moving average smoothing of the preprocessed EMG data [31]. Thus, post-processing was conducted by additionally applying an exponential moving-average filter (EMA) that reacts relatively fast and does not introduce a systematic overshoot in its step-response, compared to a widely used Butterworth post-processing filter. The EMA filter is given as:

$${\hat{y}}^{'}(t)\;=\;(1-\gamma ){\hat{y}}^{'}(t-1)+\gamma \hat{y}(t),$$ (3)

where *γ* is the filter-constant that controls the amount of smoothing. We used a velocity control modality which is generally used in prosthetics, and can be seen as another post-processing step:

$$\hat{z}(t)\;=\;\hat{z}(t-1)+{\delta \hat{y}}^{'}(t),$$ (4)

where *δ* is the velocity coefficient that determines the movement velocity of the cursor and **ẑ**(*t*) is the final cursor position in 2D space, controlled by the users’ EMG in real time. Since the two parameters interplay together to control the smoothness of the cursor and its speed, a proper combination of those two parameters should be determined. Based on the results of the preliminary study (see section 3.1.), both system parameters were determined to 1/25, with which none of the subjects perceived a delayed system response. Also, the post-processed final regression output **ẑ**(*t*) was restricted not to exceed the limitation of the unit-circle. If the final output **ẑ**(*t*) exceeded the boundary of the unit-circle, the position of the cursor was restricted to the circle boundary.

### 2.4. Experimental protocol and procedures

#### 2.4.1. Calibration run

In the calibration run, a visual synchronization training approach was employed to acquire training data X and Y and a linear regression model **W**, where the subject was instructed to perform wrist contractions based on the position of moving visual targets. The training data X and Y consisted of the RMS features extracted from each electrode and their corresponding positions of visual targets, respectively. Because it was confirmed in our previous study [19] that a high decoding accuracy of combined wrist movements of two DoFs can be obtained by a linear regression model trained with single DoF movements, only single motions of each DoF were used in the calibration run. Fig 2(a) shows a screenshot of a calibration run, where the subject was prompted to make wrist contractions based on the position of the larger circle moving from the center (rest position) to the end point of the outer circle (full contraction). The four directions of the two wrist DoFs (flexion-extension and radial-ulnar deviation) were presented once, during which both EMG signals and target traces were concurrently recorded. The time used for each direction is 8 s.

#### 2.4.2. Online evaluation run

In the online evaluation run performed for investigating the impacts of arm position change and donning/doffing, the subject performed a target acquisition task where the subject controlled the vectorial velocity of a cursor based on muscle contractions to achieve targets. To acquire a target, the cursor should be entered into the circle within a time limit (5 s for the preliminary experiment and 10 s for the main experiment) and stayed for 1 s. There is a 1-s pause between targets for the subject to prepare the next acquisition task. Each evaluation run consisted of 16 circular targets with a radius of 0.15 units. Fig 2(b) shows the location of all 16 targets, which was fixed but randomly presented for each run and each subject.

#### 2.4.3. Preliminary experiments

**Fig 3:**
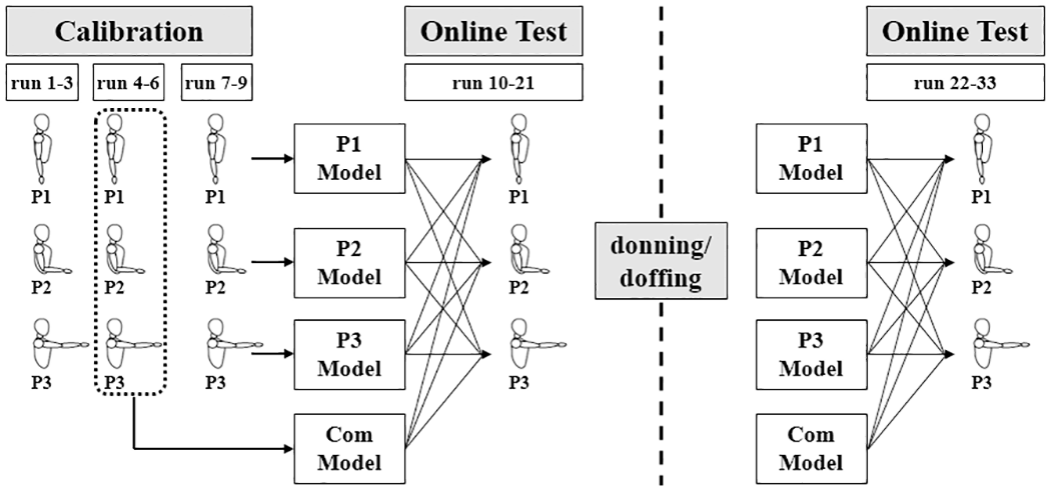
Experimental paradigm designed for investigating the impacts of arm position change and donning/doffing on the online performance of myoelectric control. Three calibration runs are sequentially performed on each arm position (P1→ P2→ P3) and this procedure is repeated three times (run 1–9). Then, three position-specific and one combinatory regression models are separately constructed by using the calibration data sets measured on each arm position and by combining one run of each arm position (run 4–6) (P1 Model, P2 Model, P3 Model, and ‘Com’ Model). Note that the same amounts of calibration data are used for modeling the four linear regression estimators to avoid biased results (3 calibration data for each model). In the online evaluation run, all possible combinations of the four regression models and three arm positions are tested using the target acquisition task (run 10–21), where the regression models and arm positions are randomly paired and presented to each subject. While the information of the current arm position is presented to the subject in the top right of the monitor screen (see Fig 2(a)), the given regression model is blinded to not only the subject but also the experimental instructor so as to prevent possibly biased results (double-blind study). After the first 12 online test runs (run 10–21), the electrode integrated hose is taken off and again put on around the original position without specific criterion to simulate a natural donning/doffing situation, and then the same 12 test runs are performed with another randomized order of the regression models and arm positions (run 22–33). The original and shifted locations of the electrodes are manually measured and recorded to see the effect of shift distance on online performance, for which the electrode locations are marked with a water-based sign pen and the distance between the original and shifted electrode locations is estimated with a tapeline. Note that electrode positions are clearly imprinted on target muscles because the electrode integrated hose naturally presses the muscles during the experiment (no subjects claimed any pain).

The preliminary experiments were conducted to determine the two post-processing parameters of the online myoelectric control system (filter constant and velocity coefficient). The subject sat on a comfortable chair facing a computer monitor with a distance of about 1 m, and wore the electrode integrated hose. A break was given whenever the subject wanted between experimental runs. Since the goal of the preliminary study was to determine the post-processing parameters, only one arm position was employed (elbow flexed at 90°, ‘P2’ denoted in Fig 3). As both the EMA filter and the velocity controller receive one sample in each system update step, their parameters depend on the system update rate *f*_*su*_(25 Hz in this study). Four values with logarithmic scaling (0.5/*f*_*su*_, 1/*f*_*su*_, 2/*f*_*su*_, 4/*f*_*su*_) were empirically selected as potential candidates and all 16 combinations were tested in the preliminary experiment. One calibration run was conducted on P2 position for modeling a linear regression estimator, and then sixteen online acquisition test runs were performed with the different combinations of the two system parameters. The order of the sixteen parameter combinations was randomized for each subject.

#### 2.4.4. Main experiments

The main experiments were conducted to evaluate the effect of arm position change and donning/doffing on online myoelectric control performance under the same condition with the preliminary experiments. Three different arm positions were used to test the impact of limb position change. Fig 3 shows the experimental paradigm used in the main experiment, where each arm position is denoted as P1, P2 and P3. In the calibration session, three calibration runs were sequentially performed on each arm position (P1→ P2 → P3), and this procedure was repeated three times (run 1–9). Then, three position-specific regression models were separately constructed using the calibration data sets measured on each arm position (P1 Model, P2 Model and P3 Model in Fig 3). Another regression model was also constructed by combining one run of each arm position (run 4–6) (‘Com’ Model in Fig 3). The reason for use of the second iteration of the three calibration runs is that it is assumed that using either the first (run 1–3) or the third (run 7–9) iteration would result in biased regression models because the subject is less or more familiar with the experimental paradigm. In fact, there were no statistically significant differences between the training performances of the four regression models, which were estimated with the multivariate R^2^ index according to the below (Eq 5) (Friedman test; P1: 0.69 ± 0.06, P2: 0.66 ± 0.05, P3: 0.68 ± 0.05, Com: 0.66 ± 0.04). The ‘Com’ Model is introduced because some studies based on pattern recognition approaches reported that a classifier trained with calibration data acquired from multiple arm positions can prevent significant performance drop when test arm positions are changed, compared to using single position calibration data [43–45]. In the online test runs, the target acquisition task was performed for all possible combinations of the four regression models and three arm positions. Donning and doffing was performed after the first twelve online runs, and the same online test runs were repeated.

### 2.5. Performance metrics and statistical analysis

Offline regression accuracy was evaluated using the calibration EMG data (run 1–9) to check the impact of arm position change on offline performance for which a three-fold cross-validation was applied [53]. In every cross-validation step, two of three calibration runs for each position were used for training the linear regression models of each position, and the other remaining run for the same position and all three calibration data for different positions were used for estimating the offline performance of intra- and inter-arm positions. For a combined regression model, one iteration of each position (i.e. run 1–3, 4–6 or 6–9) was used for training, and the other two calibration runs measured from each position were used for evaluating the offline performance of the combined model for each position. This procedure was repeated three times by alternating the calibration data set. Note that because a combined model was built with more training data (3 runs) than individual position models (2 runs), there might be the possibility that the performance of a combined model is generally higher than those of individual ones. However, the aim of offline analysis was to see the relative performance change with respect to different test arm positions on a fixed training position; we note that the performance difference between regression models did not significantly affect the main results of the offline analysis. Offline estimation performance was calculated with the multivariate R^2^ index [54], which has been widely used to estimate the performance of regression algorithms:

$$R^{2}\;=\;1-\;\frac{\sum_{d}Var(y_{d}-{\hat{y}}_{d})}{\sum_{d}Var(y_{d})},\;$$ (5)

where **y**_*d*_ and **ŷ**_*d*_ indicate the reference cursor positions of the *d*-th DoF (the position of the larger circle presented in the calibration session) and cursor positions estimated by the trained linear regression model, respectively. The numerator in the second term is the total mean square error, which is normalized by the variance of the reference cursor positions. Therefore, the maximal r-square value is one in case that the trained linear regression model completely predicts the true cursor positions. A negative r-square value is also possible when the prediction error is larger than the variance of the reference cursor positions.

To choose the two post-processing parameters (filter constant and velocity coefficient), two performance metrics were considered, e.g., completion rate and completion time. For the main experiment, additional five quantitative performance metrics were employed to assess various aspects of real-time myoelectric controllability, thereby comprehensively investigating the effect of arm position change and donning/doffing. The description of all seven performance metrics are given in Table 1. For the throughput metric, the task difficulty index (TDI) was defined as a function of the target distance because the same width was used for every target:

$$TDI\;=\;{log}_{2}(1+D),$$ (6)

where D is the target distance from the center in the GUI interface, and throughput was finally calculated by dividing completion time from TDI. Even though throughput contains the information of completion time, throughput and completion time were separately reported in this study because throughput cannot directly represent completion time. Among the seven performance metrics, user effort is introduced as a measure of effort to complete the target acquisition task. Since the RMS feature used in this study has been used for evaluating user effort in previous EMG studies [55–57], we also used the mean RMS value averaged over all recording channels and the whole experimental time to calculate the user effort for each experimental run.

**Table 1:** Description of seven performance metrics.

Metric	Description
Completion Rate (%)	Ratio of successfully achieved targets to the total number of targets
Completion Time (s)	Mean time taken achieving a target; the time limit of 10 s was counted for missed targets
Throughput (bit/s)	Ratio of the task difficulty index (TDI) and the completion time; information transfer rate defined by Fitt’s law [58]
Path Efficiency (%)	Ratio of the shortest path between a target and the initial (center) position to the actual path travelled
Overshoot (%)	Ratio of the number of times a target is reached but left before 1 s dwell time to the total number of targets
Stopping Path (travel length)	Path length travelled in a target circle for 1 s dwell time; it is applied only for achieved targets
User Effort	Ratio of the mean RMS value measured during each online test run to that averaged over the calibration runs

To check how significantly the impacts of limb position change and donning/doffing influences myoelectric control performance, non-parametric statistical tests, Friedman and Wilcoxon signed-rank, were performed because all testing data sets were not normally distributed which was assessed by the Kolmogorov-Smirnov test. The Friedman and Wilcoxon signed-rank tests correspond to the parametric statistical tests, one-way repeated measures ANOVA and paired t-test, respectively. In case of testing significant differences between more than three groups (e.g, comparing performance values of three test arm positions obtained from a fixed training position), the Friedman test was first applied to reveal overall differences between the groups. When the result of the Friedman test showed the overall significant difference between the related groups, the Wilcoxon signed-rank test was then separately performed on all possible combinations of two groups as post-hoc analysis to pinpoint which groups differ from each other in which a Bonferroni-adjusted significance level was used, i.e., *p* = 0.05/the number of post-hoc tests. To compare two groups for a statistical difference (e.g., comparing two mean performance values obtained before and after donning/doffing), the Wilcoxon signed-rank test was simply conducted. Besides the statistical tests, a Pearson correlation analysis was also performed to examine the relationship between offline and online performance in terms of arm position change, and between electrode shift distance and online performance change, respectively. The significant level for all statistical tests was set to 0.05. The data of the subject with congenital upper-limb deficiency was not included in the statistical tests, but independently analyzed as a case study.

## 3. Results

### 3.1. Results of preliminary experiments

**Fig 4:**
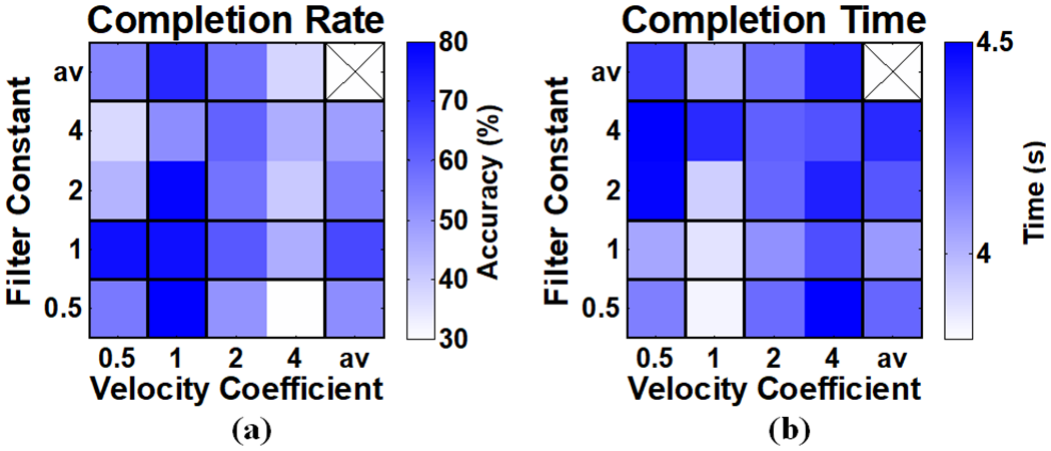
(a) Completion rate and (b) completion time calculated during the preliminary experiments. The label, ‘av’, denotes ‘average’, and the first row and last column show the average performance obtained using the corresponding parameter values. The best mean completion rate and time are obtained when 1/25 is used for the both parameters, considering the average performance of each parameter value. Also, the subjects who participated in the preliminary experiments mentioned more stable controllability with a combination of 1/25 filter constant and 1/25 velocity coefficient. Note that the values in x- and y-axis are normalized by the update rate (25 Hz).

Fig 4 shows the mean resulting matrices of completion rate and completion time with respect to velocity coefficient and filter constant, where the first row and last column (denoted by ‘av’) represent the average performance for each testing value of velocity coefficient and filter constant, respectively. The two performance metrics show the best results when 1/25 is used for both velocity coefficient and filter constant, considering the mean performance of each parameter value. Thus, the two post-processing parameters were set to 1/25 through the whole experiments, as mentioned in the section 2.3.4.

### 3.2. Impact of arm position change and donning/doffing

#### 3.2.1. Offline results with respect to arm position change

**Fig 5:**
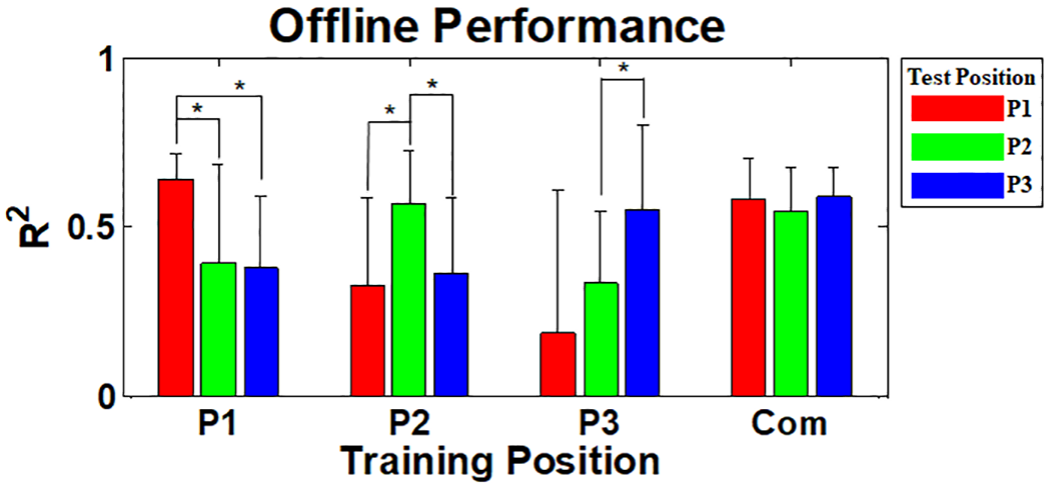
Mean intra- and inter-position offline performance of the thirteen able-bodied subjects. The red, green, and blue bars show the mean R^2^ values estimated from three test arm positions (P1, P2, and P3), respectively, for the corresponding training positions (P1, P2, P3, and Com). The mean intra-position R^2^ values are significantly higher than the inter-position ones at all training positions, and there are statistical differences between them for most cases (*p* < 0.05). Good offline performance is attained from all test positions at the ‘Com’ training position and there is no considerable performance difference between test positions. The error bars represent the standard deviations of the mean R^2^ values.

Fig 5 shows the mean inter- and intra-position offline performance over all able-bodied subjects, where the red, green and blue bars represent the offline performance of three respective test positions (P1, P2 and P3) for each training position (P1, P2, P3, and Com). The mean intra-position R^2^ values are significantly higher than the inter-position ones at all training positions (*p* < 0.05). On the other hand, good offline performance is attained from all test positions at the ‘Com’ training position (*p* > 0.05).

#### 3.2.2. Online results with respect to arm position change and donning/doffing

**Fig 6:**
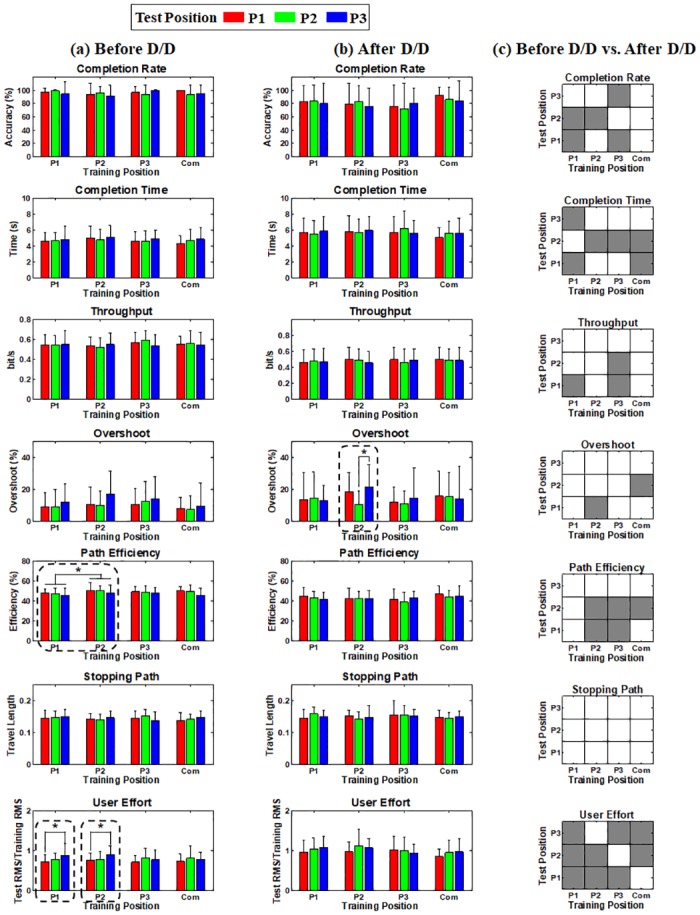
Online performance estimated (a) before and (b) after donning/doffing for all metrics, and (c) statistical test results showing the significant difference (gray colored entry, *p* < 0.05) between the performance obtained before and after donning/doffing at the corresponding combinations of training and test positions. The error bars in (a) and (b) represent the standard deviations of the performance values of the thirteen normally limbed subjects. No significant performance loss is observed even when test arm positions are different from training positions, compared to the performance obtained from the same training and test positions (a) before donning/doffing. Only two cases show significant performance differences between test arm positions (P1 and P2 training positions for user effort before donning/doffing). When comparing the online performance in terms of the training position, there is only one case showing statistically better performance than the others (P2 > P1 for path efficiency before donning/doffing). A similar trend is also observed (b) after donning/doffing even if overall online performance decreases, compared to before donning/doffing. (c) Statistically significant performance loss is frequently observed between before and after donning/doffing, except one performance metric (stopping path).

Fig 6(a) presents the mean online performance obtained before donning/doffing for the seven performance metrics, thereby showing the impact of arm position change solely when real-time feedback is provided. In general, high online performance is achieved regardless of test arm positions from all training positions (e.g., > 90% completion rate). Interestingly, contrary to the offline performance, there is no significant performance loss even when test arm positions are different from training positions, compared to the performance obtained from the same training and test positions. The little impact of arm position change on online performance is also confirmed after donning/doffing in Fig 6(b), even if overall online performance decreases. Fig 6(c) presents the statistical test results showing the impact of donning/doffing for each performance metric, where the gray colored element indicates that the online performance significantly decreases after donning/doffing at the corresponding combinations of training and test positions. Significant performance drop is frequently observed in all performance metrics after donning/doffing.

**Fig 7:**
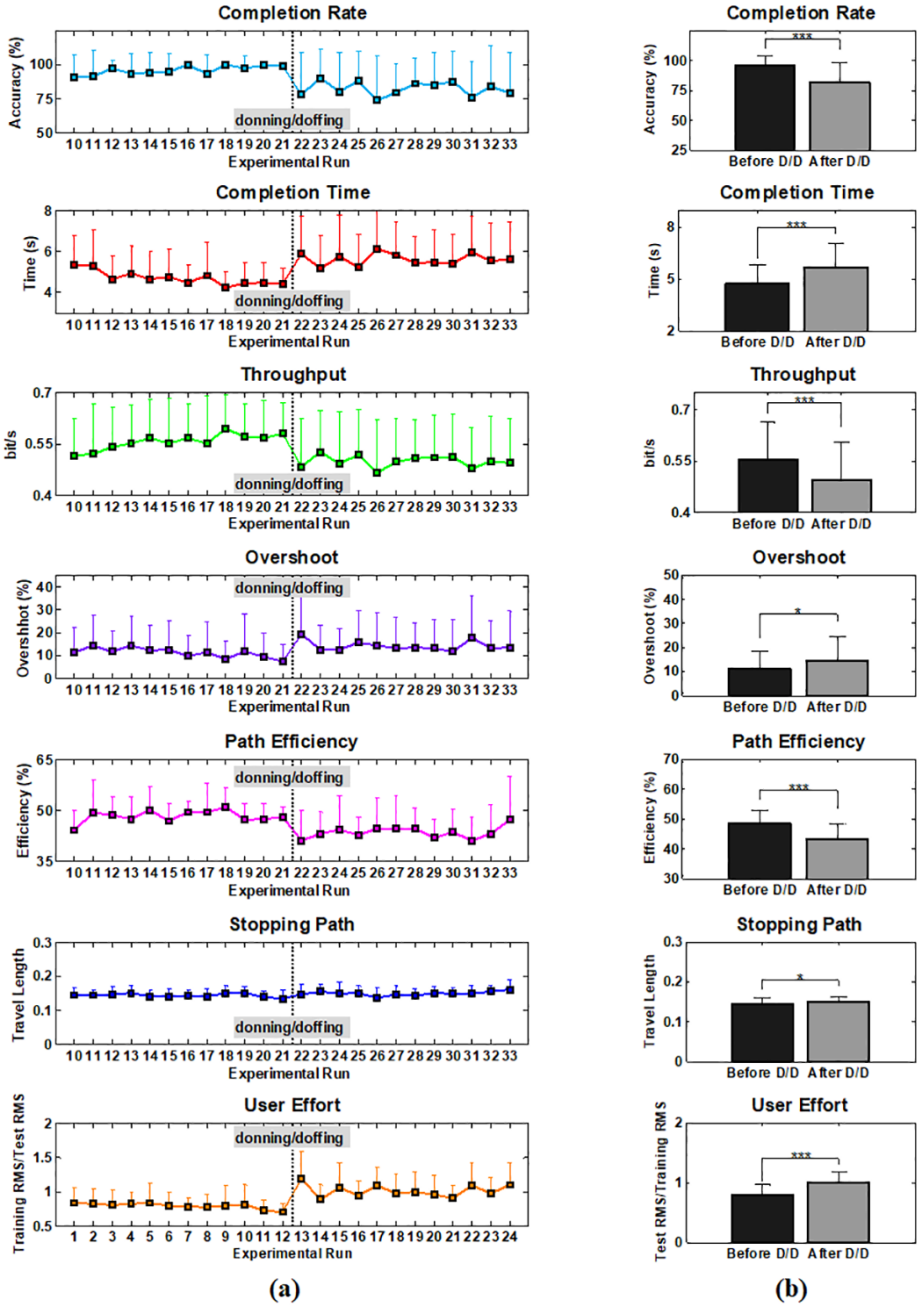
**(a) Online performance estimated according to the sequence of online evaluation runs for each performance metric (run 10–33)**. Note that due to the subject-specific randomization different combinations of training and testing arm positions were averaged, and thus the general impact of donning/doffing and growing user-experience on control performance can be observed. (b) Comparison of the mean performance estimated before and after donning/doffing (*p < 0.05 and ***p < 0.001). The error bars represent the standard deviations of the mean performance values of the thirteen able-bodied subjects.

**Fig 8:**
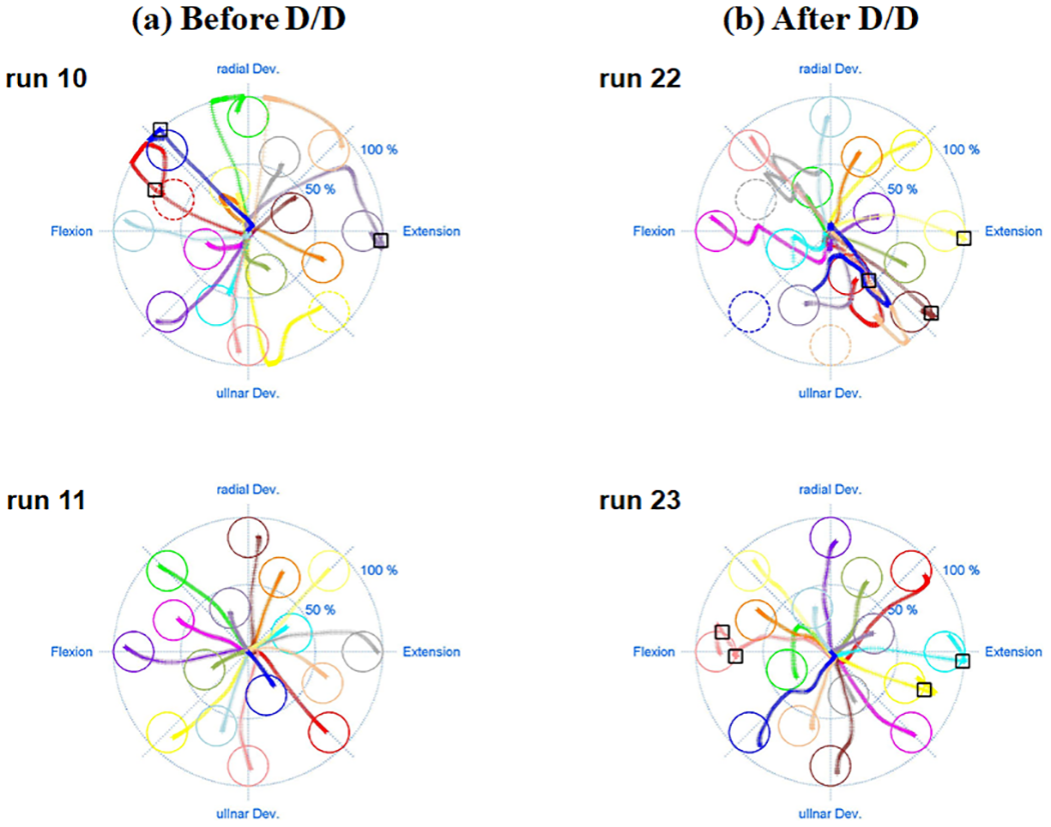
Example of cursor traces travelled during the first two evaluation runs (a) before (run 10 and 11) and (b) after donning/doffing (run 22 and 23), which are derived from the same subject. Different colored circles and traces indicate randomly presented 16 targets and the paths travelled to achieve the corresponding targets, respectively. The numbers in each target represent the sequence of target appearance. The solid and dashed circles mean successfully achieved and missed targets, respectively, and the small black rectangles represent overshoot. The mean path efficiencies of run 10, 11, 22, and 23 are 40.40, 62.16, 40.75, and 46.29%, respectively.

Fig 7 is another result that illustrates the impact of donning/doffing on the online control performance in which Fig 7(a) shows the performance of each metric in the sequence of online evaluation runs (10–33 runs). Even though differently randomized combinations of training and test positions were used for each subject during the experiments, the sequence results shown in Fig 7(a) would be reasonable to examine the general impact of donning/doffing and potential performance improvements due to growing user-experience because there was little effect of arm position change on the online performance, as shown in Fig 6. A similar trend is observed in most performance metrics: the online control performance is generally increasing as the test run proceeds until donning/doffing occurs (run10–21), rapidly deteriorated right after donning/doffing (run 22), fairly recovered in the next run (run 23), and then retained in overall (run24–33). Fig 7(b) shows the average performance attained before and after donning/doffing. It is statistically confirmed that the performance considerably decreases after donning/doffing in all performance metrics (*p* < 0.05), which is in line with the results shown in Fig 6(c). In order to visually describe these trends, a representative example of cursor traces shown during the first two evaluation runs before (run 10 and 11) and after donning/doffing (run 22 and 23) is presented in Fig 8. The subject shows somewhat unstable controllability in the first test run (run 10) especially when attempting to achieve some targets presented around the edge of the outer circle, but much more stable controllability in the next test run (run 11). The controllability gets significantly worse right after donning/doffing (run 22), but is considerably improved in the next test run (run 23). Despite of the improvement, however, the controllability is still not completely recovered as the most stable one (run 11) shown before donning/doffing.

#### 3.2.3. Relationship between offline and online results with respect to arm position change

**Fig 9:**
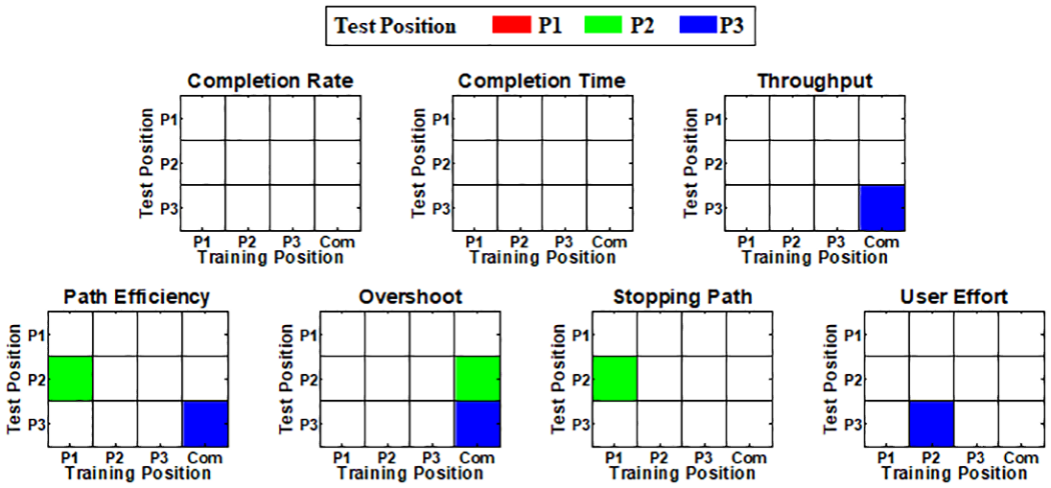
Correlation matrices showing the significant correlation (colored element) between the offline and online performance at the corresponding combinations of training and test positions. Diagonal and off-diagonal elements represent the correlation results when no impact and arm position change impact are introduced, respectively. When training and test arm positions are the same (no control condition change), there is no significant correlation between the offline and online control performance (see the diagonal entries of the correlation matrices). Only three of forty-two cases show statistically significant correlation between the offline and online performance when the impact of arm position change is introduced (see the off-diagonal entries of the correlation matrices). The correlation analysis results for the ‘Com’ training position were not considered in counting the number of the significant cases, because the ‘Com’ model was trained by combining the training data of all training positions.

Fig 9 shows the relationship between the offline and online performance respectively shown in Figs 5 and 6(a) for all possible combinations of training and test positions for each performance metric. In the correlation matrices in Fig 9, colored element represents the statistically significant correlation between the offline and online performance at the corresponding combinations of training and test arm positions. While there is no significant correlation between the offline and online control performance (see the diagonal entries of the correlation matrices) when training and test arm positions are the same, three cases show statistically significant correlation when the impact of arm position change is introduced (see the off-diagonal entries of the correlation matrices).

### 3.3. Impact of electrode shift distance

An EMG study based on classification approach reported that electrode shift caused by donning/doffing generally decreases myoelectric control performance and the degree of the performance loss highly depends on shift distance [41]. Therefore, we also investigated the impact of electrode shift distance on the control performance, for which the correlation between shift distance and the performance change triggered by donning/doffing was estimated. The performance change was estimated by subtracting the performance attained before donning/doffing from that attained after donning/doffing. Note that the shift distance of the electrodes was not significantly different among the subjects (1.16 cm ± 0.34) because we donned and doffed the textile hose including the recording electrodes in a natural way rather than restricting shift distance to precisely simulate real donning/doffing situations. Interestingly, there are no cases showing significant correlation between electrode shift distance and changes in the online performance (not shown here).

### 3.4. Results for the subject with congenital deficiency

**Fig 10:**
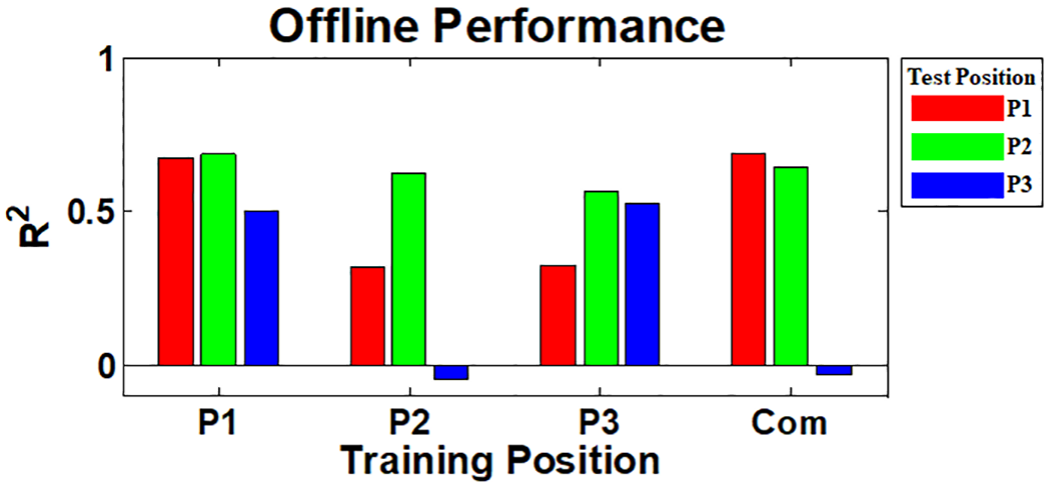
Offline intra- and inter-position performance of the subject with congenital deficiency. The red, green and blue bards show the R^2^ values estimated from three test arm positions (P1, P2, and P3), respectively, for the four training positions (P1, P2, P3, and Com). The fundamental trend shown in the able-bodied subjects’ result (Fig 5) is similarly observed, in that intra-position R^2^ values are generally higher than inter-position ones. Compared to the offline result of the intact-limb subjects (Fig 5), there are two distinct points: 1) P2 test position performs good for all training positions and 2) P3 test position is more severely influenced by the training position (see the performance of P3 test position at P2 and ‘Com’ training positions).

The offline inter- and intra-position performance of the subject with congenital deficiency is presented in Fig 10, where the red, green and blue bars indicate the offline performance obtained from respective three test positions for the corresponding four training positions (P1, P2, P3, and Com), respectively. The fundamental trend shown in the able-bodied subjects’ result (Fig 5) is similarly observed in Fig 10, in that the original offline performance attained from the same training and test position is relatively high, but that is not retained for all other arm positions.

**Fig 11:**
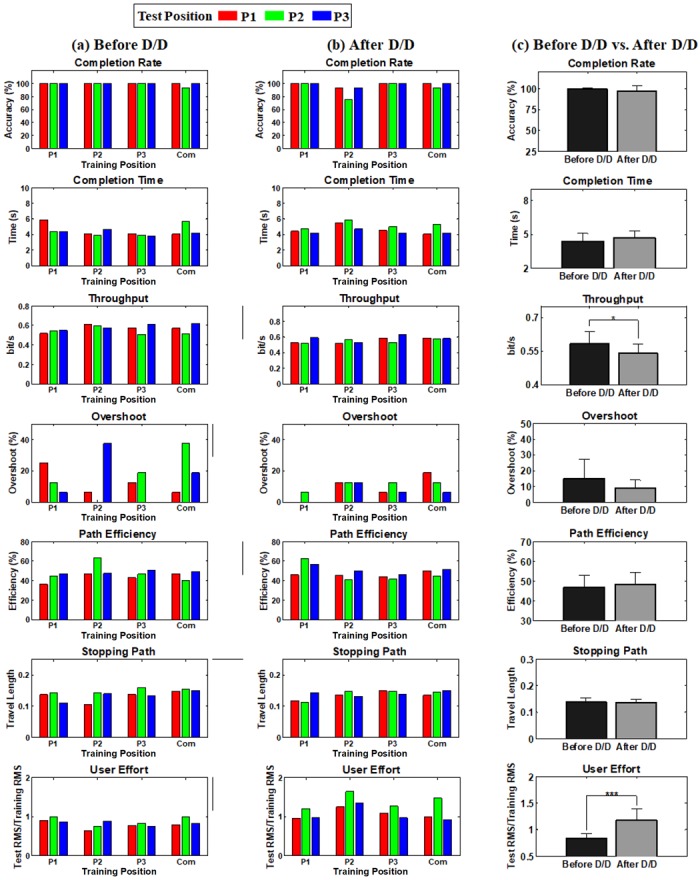
Results of the subject with congenital upper-limb deficiency. Online Performance estimated (a) before and (b) after donning/doffing for all metrics, and (c) comparison of the mean performance estimated before and after donning/doffing (*p < 0.05 and ***p < 0.001). The error bars represent the standard deviations of the performance values. Although the overall online performance decreases after donning/doffing as the results of the able-bodied subjects, the original performance obtained before donning/doffing is fairly retained even after donning/doffing. Unlike the online results of the able-bodied subjects, significant decrease in online performance is shown in only two performance metrics, throughput and user effort, after donning/doffing. Also, two other performance metrics, overshoot and path efficiency, even show slightly better performance after donning/doffing in average.

Fig 11(a) shows the online control performance of the subject with congenital upper-limb deficiency obtained before donning/doffing for each combination of training and test position, respectively, for all performance metrics. Similar to the online results of the able-bodied subjects shown in Fig 6, high control performance is seen even when test positions are changed (e.g., 100% completion rate for most cases), and the original performance obtained before donning/doffing is fairly retained even after donning/doffing (Fig 11(b)). This trend is also confirmed in Fig 11(c) showing the mean online performance estimated before and after donning/doffing, respectively.

**Fig 12:**
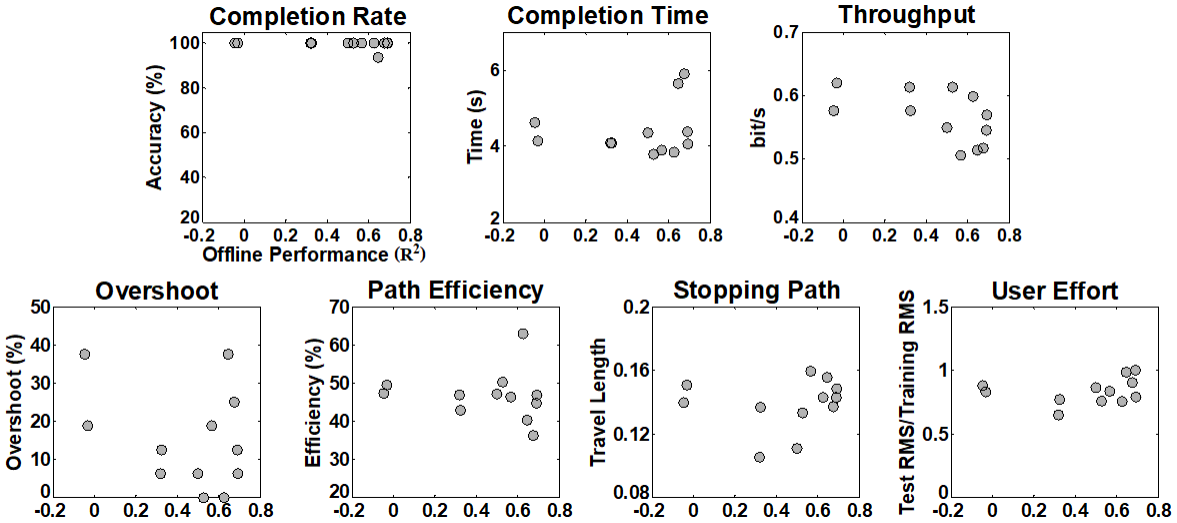
Correlation between offline and online performance obtained from the subject with congenital deficiency. The circles in each panel indicate online performance against offline performance for all twelve combinations of training and test positions. No statistically strong correlation is found for each performance metric.

Fig 12 shows the statistical relationship between the offline and online performance for each performance metric, where the gray circles in each panel represent online performance against offline performance for all twelve combinations of training and test positions. It is clearly observed in Fig 12 that the congenial subject can achieve high online performance irrespective of offline performance, and thereby there is no case showing statistically significant correlation between the offline and online performance.

The electrode shift distance occurred during donning/doffing is 1.12 cm, which is similar to the mean shift distance of the able-bodied subjects (1.16 cm ± 0.34).

## 4. Discussion

Myoelectric control algorithms for multifunction hands have been extensively studied and substantially advanced, but as indicated above, most commercially available prostheses are still operated by the direct control method proposed about fifty years ago [3]. This is because several practical factors significantly deteriorate the reliability of the current-state-of-art of control algorithms, and further restrict the clinical applicability of myoelectric control. Two of the most important factors are the changes in arm postures and electrode locations that often occur during the daily use of prostheses. To tackle the reliability problem associated with these two factors, many studies examined how they affect myoelectric control performance [20, 21, 40–46, 51]. However, unfortunately, the effect of the clinically relevant factors has been mostly studied in offline experimental settings that fundamentally do not reflect practical myoelectric control environments, where the user and a myoelectric controller interplay with each other. Only few classification studies examined the effect of channel shift [40, 41] and arm position change [47] on real-time myoelectric controllability, but the impact of the respective condition change has rarely been investigated in online control environments for regression-based approaches. Furthermore, no studies have investigated the combined impact of arm position change and electrode shift mainly caused by donning/doffing together neither in offline nor online settings even though they generally occur simultaneously during use of a prosthetic hand. In this study, we investigated the two factors independently and concurrently in practical closed-loop control scenarios to precisely characterize the impacts of the two sources of non-stationarities.

The strong negative impact of arm position change on offline performance that was shown in previous studies [21, 43–46] was also confirmed in our offline experiments (Figs 5 and 10), meaning that the applied linear regression algorithm itself was not robust to arm position change similar as previously investigated pattern recognition methods. However, the online control performance did not significantly change between different arm positions when real-time feedback was provided, even when training and test positions were mismatched (see Figs 6(a) and 11(a)). The retained online performance can be explained by the fact that the user can overcome the adverse impact of arm position change by interacting with a myoelectric control system if instantaneous real-time feedback is provided. This indication could also explain that there was little advantage in using the ‘Com’ model trained by a combination of all training positions over the other models trained from each single position (Figs 6 and 11) when tested in online control condition. Because of the different tendency in the experimental results, significant correlation between the offline and online performance was observed in only few cases (Figs 9 and 12), inferring that offline performance cannot accurately predict online control performance. Even though there may exist other offline performance metrics that do correlate with online performance, our study is an example that this is not generally the case, as also found in other recent studies [48, 59].

On the other hand, donning/doffing was still problematic even in online control environments (see Figs 6(c), 7(b) and 11(c)), which means that the adverse impact of donning/doffing was too strong to be overcome even with real-time feedback, in contrast to that of arm position change. Nevertheless, real-time feedback might still play a role in partly compensating the negative effect of donning/doffing on the control performance (Fig 7). In particular, the congenital subject showed less impact to donning/doffing in terms of online performance (Fig 11), showing the importance of real-time feedback.

The online performance significantly decreased after donning/doffing, but did not decrease considerably further after the impact of arm position change was additionally introduced (see Figs 6(b) and 11(b)), which is another indication of little impact of arm position change on real-time myoelectric controllability. Thus, it can be thought that arm position change is of lesser critical concern in practical control situations, but mechanical or algorithmic solutions are needed to resolve the negative impact of donning/doffing. Future work for alleviating the negative impact of donning/doffing could be the development of a well fitted prosthetic socket which heavily depends on the skills and the experience of the orthopedic technician, and novel control algorithms that are more robust to related non-stationarities.

Although our control system has shown a high robustness against changing arm position when tested online, other posture related factors may still impact the performance. According to recent studies [60–62], changing wrist positions can significantly deteriorate both offline and online myoelectric control performance for able-bodied subjects and partial-hand amputees. Therefore, further work should be conducted to investigate related factors in more detail and for other user groups such as trans-carpal amputees.

User effort was the only index significantly affected by the change of arm position (Fig 6(a)), and the most sensitive to the donning/doffing impact in both able-bodied subjects (Fig 6(c)) and the subject with congenital deficiency (Fig 11(c)). This indicates that the user indeed adapted his/her EMG activation patterns to changed control environments in order to compensate them, even if this was fully successful only for arm position change. The stronger muscle contractions reflected in the increased user effort may lead to an increased muscle fatigue in long-term use of prostheses. Thus, user effort first introduced for myoelectric prosthesis control in this study could be considered as a useful index in developing clinical myoelectric prostheses along with the conventional performance metrics directly inferring myoelectric controllability (e.g., completion rate and time).

In this study, donning/doffing was performed in a natural way so that a realistic donning/doffing can be simulated as in clinical control situations. The mean distance of electrode shifts was approximately 1 cm (1.16 cm ± 0.34) which is within the same range that occurs in clinical practice [41]. Even though donning/doffing caused a substantial decrease in myoelectric controllability, there was no strong correlation between electrode shift distance and control performance. This could be partly caused by other factors, besides electrode-shifts, such as changed impedances, or potential correlations might not be detected due to the relatively small variations in shift-distance presented in the naturally applied donning/doffing procedure. Also, a non-linear behavior such as a step-like drop in performance at lower shift-distances could be the reason for the lack of any significant correlation. Although out of the scope of this study, future work could investigate these aspects in more detail.

One subject with congenital upper-limb deficiency participated in this study, whose overall results were similar as in case of the able-bodied subjects. This indicates that prosthetic end-users can also adapt to changing myoelectric control conditions similar as able-bodied subjects when real-time feedback is provided. This is in line with a recent online study showing no significant difference on all performance metrics between amputees and able-bodied subjects [30]. Traditionally, it has been accepted that the results obtained from intact-limb subjects can be fairly transferred to persons with amputation even though the absolute performance of amputees is somewhat lower than that of able-bodied subjects [4]. Thus, although the results of only one congenital subject were shown in this study, it is highly possible that our main outcomes underlined in this study apply to the majority of amputees and subjects with congenital limb deficiency.

In summary, the effect of arm position change and donning/doffing were systematically investigated in online control environments to accurately understand how the both condition changes affect myoelectric controllability. The main findings of this study are as follows: (1) changes in arm positions significantly affect offline performance as shown in previous literatures, but are nearly negligible in online control conditions regardless of donning/doffing, thereby showing no correlation between offline and online performance, (2) donning/doffing significantly influences myoelectric online control performance, as already shown in several previous offline studies, (3) a model trained using multiple position data does not give a specific benefit over the single position models when applied online, (4) there is no significantly better training position than the others with respect to myoelectric controllability, (5) user effort can be used as a practical index to quantitatively estimate the risk of user fatigue, and (6) distance of electrode shift caused by donning/doffing is not significantly related to the magnitude of performance drop within our experimental settings. Except the finding (2), the other results provide new insights into robust myoelectric control, and in particular some of them are conflicting with previously reported results (findings (1) and (3)), which would be caused by different control conditions (offline without instantaneous feedback vs. online with real-time feedback). In conclusion, in future studies all clinical factors involving robust myoelectric control should be investigated in real-time under practical control conditions to precisely define their impact on myoelectric controllability. This will ultimately permit to find better and more practically robust solutions for the clinical setting.
